# Editorial: AI, sensors and robotics in plant phenotyping and precision agriculture, volume II

**DOI:** 10.3389/fpls.2023.1215899

**Published:** 2023-06-05

**Authors:** Daobilige Su, Yongliang Qiao, Yu Jiang, João Valente, Zhao Zhang, Dongjian He

**Affiliations:** ^1^ College of Engineering, China Agricultural University, Beijing, China; ^2^ Australian Institute for Machine Learning (AIML), The University of Adelaide, Adelaide, SA, Australia; ^3^ Horticulture Section, School of Integrative Plant Science, Cornell University, Geneva, NY, United States; ^4^ Information Technology Group, Wagenigen University & Research, Wageningen, Netherlands; ^5^ Key Laboratory of Smart Agriculture System Integration, Ministry of Education, China Agricultural University, Beijing, China; ^6^ Key Laboratory of Agriculture Information Acquisition Technology, Ministry of Agriculture and Rural Affairs of China, China Agricultural University, Beijing, China; ^7^ College of Mechanical and Electronic Engineering, Northwest A&F University, Yangling, Shaanxi, China

**Keywords:** sensors, robotics, plant phenotyping, precision agriculture, artificial intelligence

## Introduction

With climate change and population growth, the ratio of food production to demand is increasingly shrinking. Plants and their production are crucial for retaining the sustainability for the natural ecosystem and human food security (Qiao et al., 2022). Rapid development and technology progress in robotics and artificial intelligence (AI), plant phenotyping and precision agriculture start to play an important role in intelligent plant phytoprotection, soil protection, reducing chemicals and labor cost, and ensuring food supply (Qiao et al., 2022). Plant phenotyping refers to obtaining the observable characteristics or traits jointly affected by their genotypes and the environment, and is formed during plant growth and development from the dynamic interaction between the genetic background and the physical world in which plants develop (Li et al., 2020).

Precision agriculture helps to maximize efficiency of soil and water usage, with the objective of minimizing loss and waste. It also increases the yield of crops, as well as reduce the variability and input costs (Cisternas et al., 2020).

**Figure 1:**
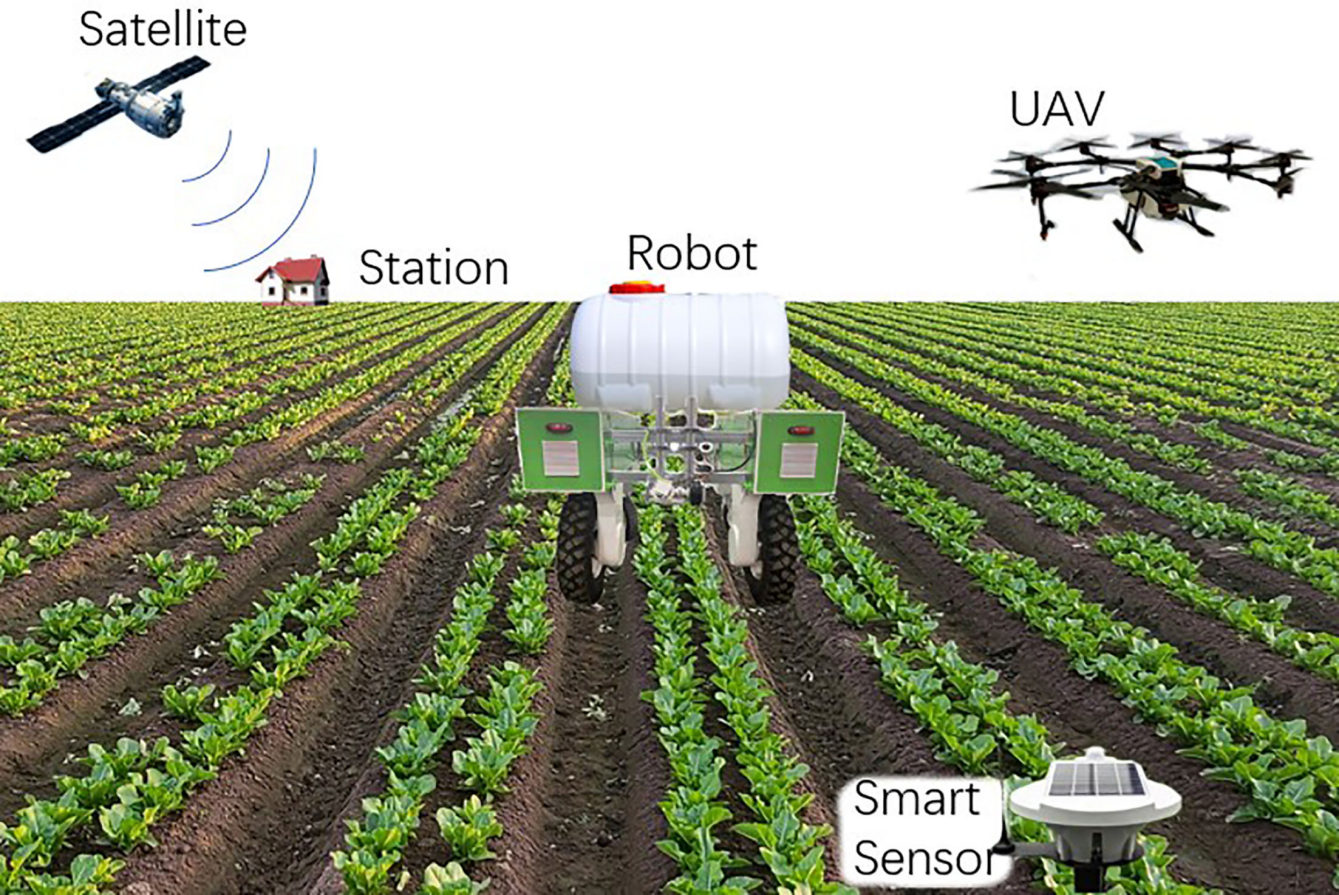
AI, sensors and robotics based dynamic 3-D plant phenotyping and precision agriculture framework.

In recent years, researchers have made a significant progress in developing various AI methods, sensor technologies and agricultural robots for planting and monitoring plants (Weyler et al., 2021; Lottes et al., 2020; Hu et al., 2022 and Su et al., 2021), as shown in **Figure 1**. A significant number of plant morphological, physiological, and chemical parameters can be rapidly and conveniently measured using AI (Li et al., 2020). Additionally, the integration of AI and robotics technologies enables real-time monitoring of plants in complex field and controlled environment (Atefi et al., 2021). By probing the complex physiology of plants through plant phenotyping, higher quality plant seeds can be obtained (Watt et al., 2020). Moreover, during plant protection processes, the application of pesticides and fertilizers can be reduced, ultimately contributing to a more sustainable agricultural environment (Vélez et al., 2023).

## Plant phenotyping

As an effective tool and process, plant phenotype is an essential part of modern, intelligent, and precise agricultural production. Various physiological and morphological parameters about plants are acquired by various sensors such as RGB cameras, lidar and multiple and hyperspectral cameras to serve as decision-making basis for real-time and future plant management (Rivera et al., 2023).

Shen et al. proposed a new backbone network ResNet50FPN-ED for the conventional Mask R-CNN instance segmentation to improve the detection and segmentation capability of grape clusters in complex field environments. The average precision (AP) was 60.1% on object detection and 59.5% on instance segmentation. Sun et al. proposed a multi-scale cotton boll localization method called MCBLNet based on point annotation, which achieved 49.4% average accuracy higher than traditional point-based localization methods on the test dataset. Based on an improved YOLOv5 model, Wang et al. proposed a fast and accurate litchi fruit detection method and corresponding software program. The results showed that the mean average precision (mAP) of the improved model was increased by 3.5% compared with the original model, and the correlation coefficient R^2^ between the application test and the actual results of yield estimation was 0.9879. Based on imaging technology, Li et al. performed three-dimensional reconstruction, point cloud preprocessing, phenotypic parameter analysis, and stem and leaf recognition and segmentation of corn seedlings in sequence, paving a new path for maize phenotype research. Li et al. proposed a Germination Sparse Classification (GSC) method based on hyperspectral imaging to detect peeled damaged fresh maize. The results show that the overall classification accuracy rate of this method in the training set is 98.33%, and the overall classification accuracy rate of the test set is 95.00%.

## Plant disease detection

Pests and diseases occur irregularly and are harmful in plant growth and production. It is critical to detect pests and diseases in time for taking necessary actions. Recent advances in computer vision makes it a popular approach to accomplish this task (Guo et al., 2023).

Aiming at the problem of rapid detection of field crop diseases, Dai et al. proposed a novel network architecture YOLO V5-CAcT. They deployed the network on the deep learning platform NCNN, making it an industrial-grade crop disease solution. The results showed that in 59 categories of crop disease images from 10 crop varieties, the average recognition accuracy reached 94.24%, the average inference time per sample is 1.56 ms, with a the model size of 2 MB. To quantify the severity of leaf infection, Liu et al.. proposed an image-based approach with a deep learning-based analysis pipeline. They utilized image data of grape leaves infected with downy mildew (DM) and powdery mildew (PM) to test the effectiveness of the method. Experimental results showed that the DM and PM segmentation accuracies in terms of mean IOU of the proposed method in the test images were more than 0.84 and 0.74, respectively.

Cotton is an important economic crop, and its pest management has always been paid attention to. Fu et al. proposed a quantitative monitoring model of cotton aphid severity based on Sentinel-2 data by combining derivative of ratio spectral (DRS) and random forest (RF) algorithms. The overall classification accuracy is 80%, the kappa coefficient is 0.73, and the method outperforms four conventional methods. In order to facilitate easy deployment of deep convolution neural network models in mobile smart device APPs, Zhu et al. use pruning algorithms to compress the models. VGG16, ResNet164 and DenseNet40 are selected as compressed models for comparison. The results show that when compression rate is set to 80%, the accuracies of compressed versions of VGG16, ResNet164 and DenseNet40 are 90.77%, 96.31% and 97.23%, respectively. In addition, a cotton disease recognition APP on the Android platform is developed, and the average time to process a single image is 87 ms with the test phone.

## Robotics and UAVs in smart farming

With the rapid development and popularization of mobile robots and unmanned aerial vehicles (UAVs), they have been increasingly deployed for agricultural applications for automated operations to avoid dangerous, repetitive and complicated manual operations (Vong et al., 2022 and Vélez et al., 2023).

Aiming at the harvesting problems faced in precision agriculture, Zheng et al. designed a robot gripper by studying the picking problem of clustered tomatoes. The results show that in the simulation environment, the gripper can smoothly grasp the medium and large tomatoes with diameter of 65∼95 mm, and all of them meet the minimum damage force condition during grasping operation. In terms of crop management such as robotic spraying and fertilization, Hu et al. proposed LettuceTrack, a multiple object tracking (MOT) method for detection and tracking of individual lettuce plant by building unique feature. The method is designed to avoid multiple spray of the same lettuce plant. In order to solve the problem of vibration deformation caused by corn harvester working, an improved empirical mode decomposition (EMD) algorithm was provided by Fu et al. to decrease noise and non-stationary vibration in the field. The results show that the proposed model could reduce noise interference, restore the effective information of the original signal effectively, and achieve the accuracy of 99.21% when identifying the vibration states of the frame.

UAVs could be used to monitor crop health, soil moisture levels, and identify areas that require irrigation or fertilization. With the use of advanced sensors and cameras, drones can capture sensing data and conduct surveys that provide farmers with valuable insights into crop growth and yield (Zhang et al., 2023). Moreover, various aspects of the guidance, navigation, and control of UAV when applied to agriculture started to be investigated to allow real-time crop management with fleets of autonomous UAVs. Huang et al. proposed a distributed control scheme to solve the collision avoidance problem in multi-UAVs systems. Numerical simulation results show that the method can effectively control multiple UAVs to complete the plant protection task within a predetermined time. Li et al. proposed a solution for field wheat lodging identification. Drones are used to obtain 3D point cloud data of wheat, which is processed with neural network to obtain the recognition result of wheat lodging. The results show that the F1 scores of the classification model are 96.7% for filling, 94.6% for maturity, respectively.

## AI and sensors in agro-ecological environment

Plant growth and agricultural production can be unstable, since they are greatly affected by their environment. A good ecological environment including forest, land and water resources is the basis of sustainable development. Researchers are paying more attention to applying artificial intelligence and sensor technology to ecological systems, and making further contributions to sustainable plant protection by sensing and monitoring ecosystem (Maharjan et al., 2022).

Zheng et al. conducted research on forest fire hazard identification methods. They proposed an improved forest fire recognition algorithm for fire recognition by fusing backpropagation (BP) neural network and SVM classifier. They constructed a forest fire dataset and tested it with different classification algorithms. The results show that the proposed method achieves an accuracy rate of 92.73%, which proves the effectiveness of the algorithm. Based on smooth channels and ecological channels with different shapes, Zhou et al. proposed a method of arming ultrasonic sensors to obtain channel flow velocity. The results show that the method simplifies the arrangement of sensors in channel flow, and improves the accuracy of the flow measurement method. The method is helpful to promote the construction of ecological channels.

## Conclusions

Sustainable agricultural development requires efforts from multiple perspectives. Human beings need to create a good ecological environment including water resources, forests and soil to ensure that plants grow in a healthy environment. A more reasonable arrangement of sensors and the use of artificial intelligence can monitor environmental changes in real time, so that farmers can make more optimum control measures. In addition, plant phenotypes will play a more important role in future agriculture, including plant breeding and plant parameter acquisition. AI and robotics technologies have been increasingly integrated into plant protection, fertilization and harvesting to pursue higher food quality and yield.

Varieties AI methods, intelligent agricultural robots and equipment have been proposed and proven to be efficient in laboratories as well as on agricultural fields. Deployment of these methods and robots during real agricultural production, while enabling the entire process at a lower cost, is upcoming challenges for both researchers and agricultural industry. Furthermore, multi-robot collaboration including ground-to-air cooperation will shape a better smart agricultural system, and build a sustainable and circular agricultural system for future farming.

## Author contributions

DS and YQ wrote the original draft. YJ, JV, ZZ, and DH reviewed and edited the paper. All authors contributed to the article and approved the submitted version.
